# Mechanical strength of the rotator cuff and cable interface: a complete histological and biomechanical study

**DOI:** 10.1007/s00276-024-03499-3

**Published:** 2024-10-23

**Authors:** Maxime Fondin, Mathieu Miroir, Raphaël Guillin, Julien Landreau, Gevorg Ghukasyan, Alain Fautrel, Mickael Ropars, Xavier Morandi, Krystel Nyangoh Timoh, Jean-Benoît Le Cam

**Affiliations:** 1 Radiology Department, Rennes University Hospital, 16 Boulevard de Bulgarie, 35200 Rennes, France; 2 Anatomy Department, Rennes Faculty of Medicine, University of Rennes 1, 35000 Rennes, France; 3 Polyclinique du Pays de Rance, Imagerie du Pays de Rance, 76, Rue Châteaubriand, 22100 Dinan, France; 4 University of Rennes, Institute of Physics, UMR 6251 CNRS/University of Rennes, Beaulieu Campus, Building 10B, 35042 Rennes Cedex, France; 5 University of Rennes, CNRS, Inserm, Biosit UAR 3480 US_S 018, France-BioImaging (ANR-10-INBS-04), Plateforme H2P2, 35000 Rennes, France; 6 H2P2 Biosit, University of Rennes, Inserm UMR 1421 Numecan, 2 Rue du Professeur Léon Bernard, 35043 Rennes, France; 7 Department of Orthopedic Surgery, Rennes University Hospital, 2 Rue Henri Le Guilloux, 35000 Rennes, France; 8 Department of Neurosurgery, Rennes University Hospital, 2 Rue Henri Le Guilloux, 35000 Rennes, France; 9 Department of Obstetrics and Gynecology, 16 Boulevard de Bulgarie, Safe CIC 1414 Thematic Team, Rennes University Hospital, 35200 Rennes, France; 10 INSERM, LTSI - UMR 1099, University Rennes 1, Rennes, France

**Keywords:** Rotator cuff, Rotator cable, Rotator cuff and cable interface, Connective tissue, Mechanical strength

## Abstract

**Purpose:**

This study sought to evaluate the biomechanical properties of the interface between the rotator cuff and the semicircular humeral ligament or rotator cable (RCa) using histological and biomechanical techniques.

**Methods:**

Out of 13 eligible cadaver specimens, 5 cadaver shoulders with an intact rotator cuff were included, 8 were excluded due to an injured rotator cuff. The histological study enables us to describe the capsule-tendon interface between the infraspinatus tendon (IST) or supraspinatus tendon (SST) and RCa, and to detect loose connective tissue layers to determine their precise location and measure their length along the interface. The biomechanical study sought to characterize and compare the mechanical strength of the IST-RCa versus SST-RCa interfaces.

**Results:**

The average thickness of the RCa was 1.44 ± 0.20 mm. The histological study revealed a loose connective tissue layer at the IST-RCa interface, a finding not observed at the SST-RCa interface. The biomechanical study showed that the rigidity of the SST-RCa interface (72.10^–2^ N/mm) was 4.5 times higher than for the IST-RCa interface (16.10^–2^ N/mm) and the average maximum forces reached were 19.0 N and 10.6 N for the SST-RCa and IST- RCa interfaces, respectively.

**Conclusion:**

The IST-RCa interface consists of a loose connective tissue layer contrary to the SST-RCa interface. In parallel, two different groups in terms of the mechanical response were identified: the IST-RCa interface group had less rigidity and ruptured more quickly than the SST-RCa interface, therefore emerging as the most vulnerable interface and explaining a potential extension of rotator cuff tears.

**Supplementary Information:**

The online version contains supplementary material available at 10.1007/s00276-024-03499-3.

## Introduction

Rotator cuff tears are a major health issue whose incidence increases with aging, with more than half of individuals in their 80 s [19]. Interestingly, intratendinous tears like delamination corresponding to a horizontal partial-thickness split of the tendon substance [5, 6, 10], whose incidence is variable, estimated at 38–82% in patients undergoing rotator cuff repair [1, 2, 7, 9, 18], are a factor of poor prognosis as they signal a deterioration in tendon quality and complicate cuff repair [2, 3, 7].

However, while a limited number of peer-reviewed publications examined the interface between the capsule and overlying tendons, in our clinical practice we noted that supraspinatus tendon (SST) and infraspinatus tendon (IST) did not have the same connection with the semicircular humeral ligament—or rotator cable (RCa)—corresponding to a crescent of capsular fibers running perpendicular to the axis of the rotator cuff [12, 13]. Indeed, Rouvière [15] showed that “the SST is closely adherent to the capsule whereas the IST one remains separated from the underlying structures by a thin layer of loose connective tissue”. Clark et al*.* [6] investigated the anatomy of SST and IST at the microscopic scale and reported that in the region of the SST and IST “the cuff was composed of five layers defined by the attachments and orientations of the fibrous elements in each of these layers”, including “loose connective tissue in which there are thick bands of collagen fibers” at the capsule-tendon interface. More recently, Michelin et al*.* [10] showed that the posterior enthesis of the IST “laid over the articular capsule” and that cleavage of this interface between the IST and articular capsule remained possible.

These observations, which were consistent with each other, clearly suggested that the mechanical properties of the capsule-tendon interface were different depending on whether the IST or SST was concerned. However, no biomechanical study has yet been conducted to characterize these properties, which explains why the link between previously mentioned histological findings [10, 15] and the difference in the mechanical properties of the interfaces has not been established. A biomechanical study is of primary importance for a better understanding of the pathophysiology of rotator cuff tears, in particular intratendinous tears or delamination.

The aim of our study was therefore to evaluate the mechanical properties of the interface between the rotator cuff and RCa using histological and biomechanical techniques.

## Materials and methods

This cadaver study approved by our institutional review board, consisted of a histological and biomechanical analysis of embalmed cadaver shoulders. The study ran from October 2019 to February 2021.

### Dissection

The anatomical data were obtained from anatomical subjects dissected at the Rennes School of Surgery (Rennes University anatomy laboratory). The university scientific committee ensured that written consent for body donation had been obtained and filed prior to death for all anatomical subjects. Our work complied with French regulations. The study was also exempt from French law pertaining to biomedical research (Huriet–Serusclat law of December 20, 1998) as no additional interventions were required.

Thirteen cadaver shoulders were isolated and embalmed in 10%-formalin solution. Shoulders were harvested from deceased Caucasian adult donors (7 women and 6 men, mean age 83.6 ± 4.7 years), who voluntarily signed a donation form before death. Signs of former treatment (e.g. scars, sutures) and an injured rotator cuff were exclusion criteria. Out of these 13 eligible cadaver specimens, 5 healthy shoulders were included (5 right; 1 woman and 4 men; mean age 82.6 ± 4.2 years) and 8 were excluded due to a severely injured rotator cuff.

All shoulders were dissected according to the following protocol:

- the skin and subcutaneous tissues were removed,
- the deltoid and trapezius muscles were detached from the clavicle and scapula,
- the clavicle was then excised from the acromioclavicular joint,
- the acromion was sawed off at its base and retracted with the coracoacromial ligament anteriorly,
- the subacromial/subdeltoid bursa was excised and all the muscles of the rotator cuff, now visible, were inspected to identify injuries,
- the teres minor, supraspinatus and infraspinatus muscles were bluntly detached from their medial attachment to the supraspinatus and infraspinatus fossa, respectively; vessels and nerves supplying muscles were cut through,
- the distal SST and IST entheses were detached, observing their interface with the capsule and RCa; note that the RCa is a thickening of the joint capsule,
- to sum up, a SST-RCa and IST-RCa group was obtained for all 5 shoulders.

### Histological study

This study sought to describe the capsule-tendon interface between the IST, SST and RCa and to detect loose connective tissue layers to determine their precise location and measure their length along the interface.

**Fig. 1:**
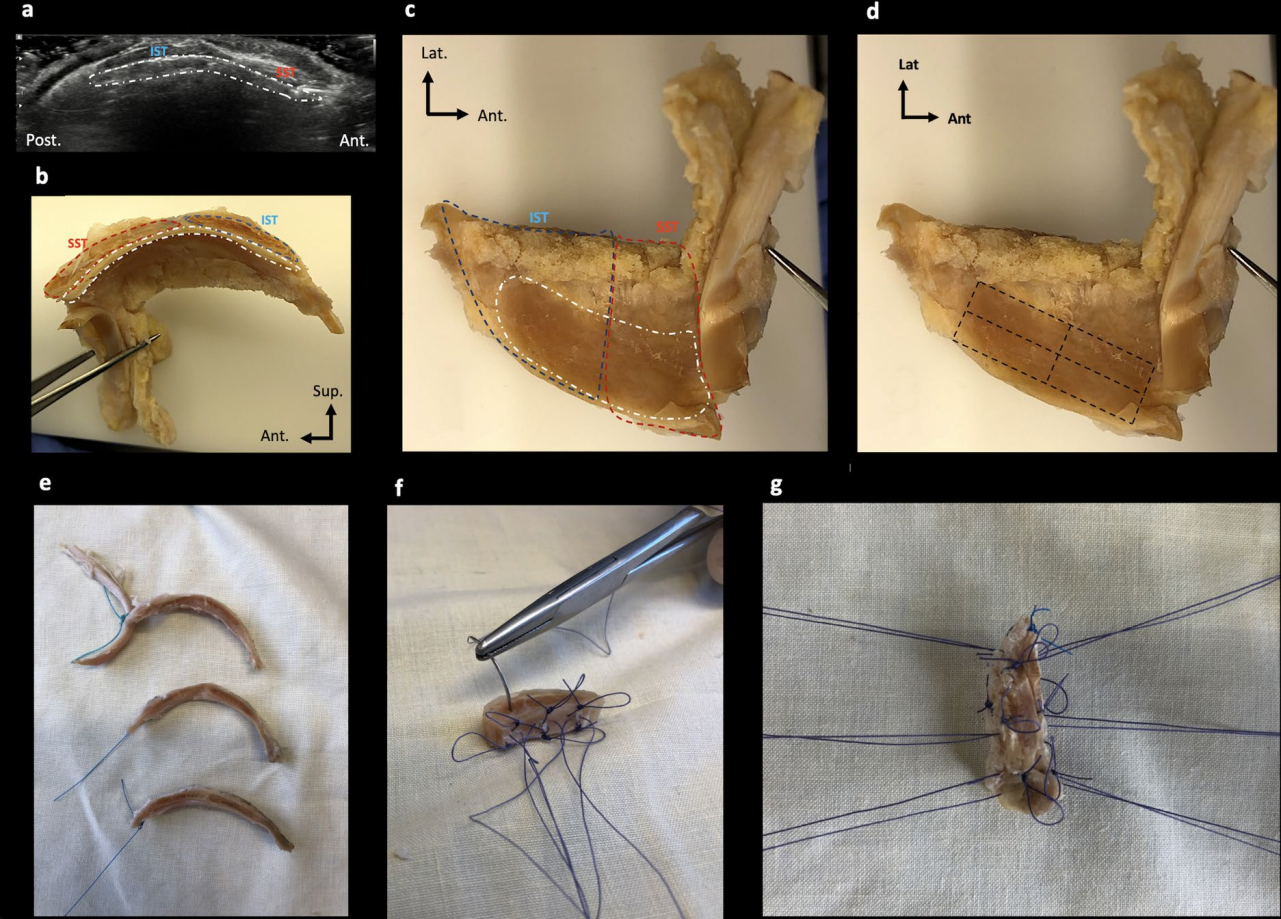
Sample preparation. **a** US imaging of sagittal section of IST, SST and RCa indicated by a white dotted line; **b, c** medial sagittal view (**b**) and bottom view (**c**) of the same sample (RCa, SST and IST in white, blue and red dotted lines, respectively); **d** boundaries of samples indicated by a black dotted line; **e** samples of comparable dimensions and interfaces were tested; **f** three hanging points were made for each tendon and the RCa with 4.0 absorbable braided thread on opposite sides; **g** final sample preparation. *Post* posterior, *Ant* anterior, *Lat* lateral

During the macroscopic examination*,* we used ultrasound (US) to orient the specimen, using anatomical landmarks such as the long biceps tendon and bicipital groove located anterior to the SST insertion, and to delineate the boundary between the SST and IST (Fig. 1).

Each group derived from the dissection protocol was first divided into 2 parts of equal thickness along the sagittal plane (the same as during US) to ensure that the histological and biomechanical studies were conducted on the same sample. Each part was then divided into 2 sub-parts to separate the IST and SST according to their respective interface with the RCa and fit into the cassettes. Note that we kept the tendon of the long biceps anteriorly as a benchmark.

Preparation of the microscopic examination included identification of samples, orientation of sections, cropping to fit cassettes (length 10 mm × width 5 mm × thickness 5 mm), and orientation in cassettes for paraffin embedding. Each paraffin block was sectioned to 4 microns thickness onto slides, and stained with hematoxylin–eosin-safran (HES). The slides were scanned with a NanoZoomer 2.0-RS digital slide scanner (Hamamatsu, Japan) at 20 × objective. Each RCa’s thickness was measured in mm in the image of the histological section with NDP.view 2 software (Hamamatsu, Japan). Three measurements were taken at the ends and in the middle of the section to determine the average thickness.

### Image processing

**Fig. 2:**
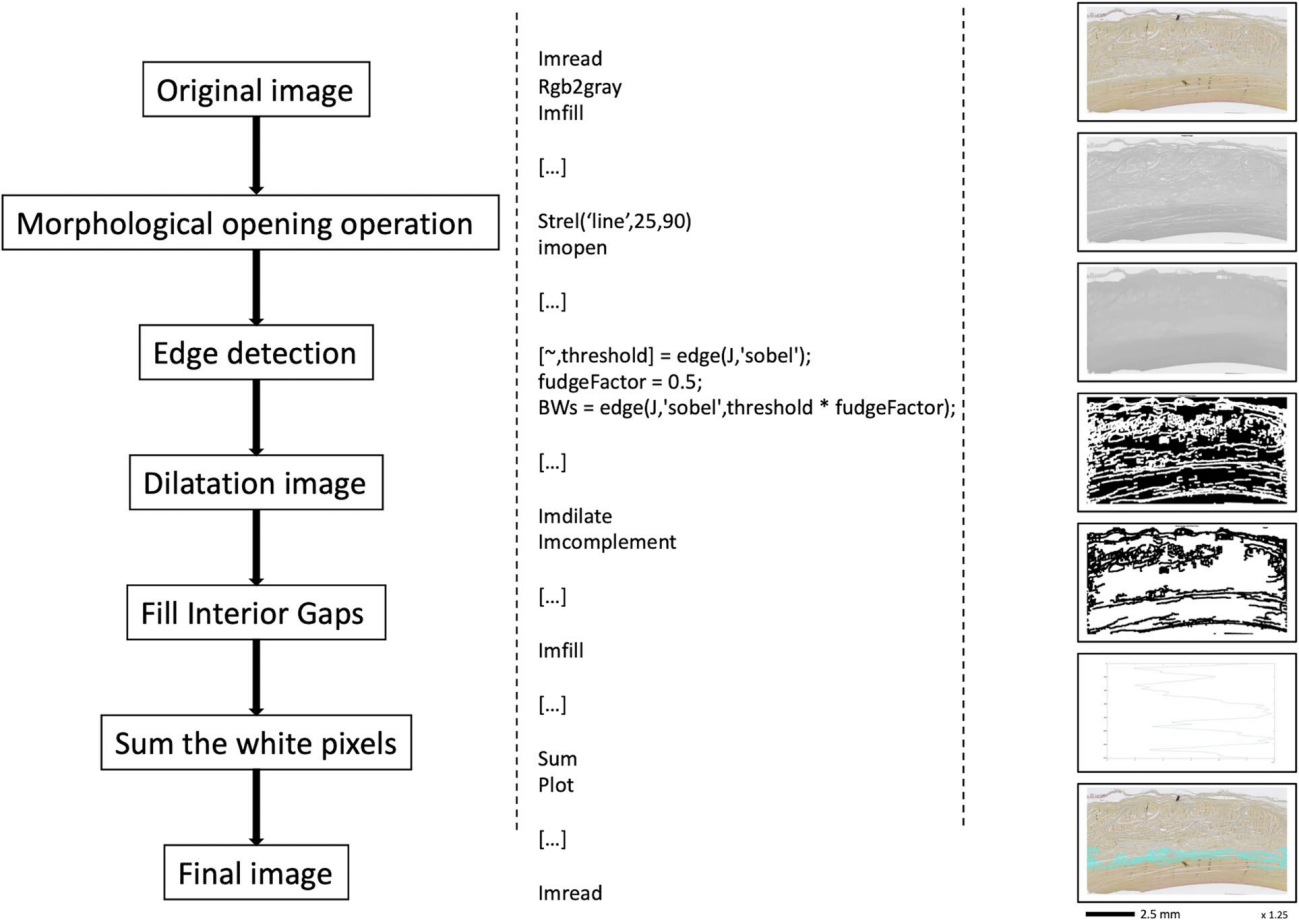
Flowchart of the basic morphological image processing algorithm

The images obtained from the digital slide scanner (Hamamatsu, Japan) were processed with NDP.view 2 software (Hamamatsu, Japan) to detect and locate loose connective tissue layers and determine their length. In addition, images were processed using Matlab software (R2017b) with the algorithm for automatic detection of loose connective tissue layers shown in Fig. 2. For this purpose, all images were reoriented and exported as TIFF (tagged image file format) files.

The next step was to detect the longitudinal patterns and contours of the sample in these images. The base image thus underwent several transformations as shown in Fig. 2. Once the image had been reworked, the number of white pixels per row was calculated and plotted. The resulting graph shows the average position of the sample contours in the image as well as the number of areas without longitudinal patterns detected. When the correct number of areas was detected, the final image was created by superimposing the detected area where the semicircular humeral tendon boundary was located over the base image.

### Biomechanical study

The biomechanical study sought to characterize and compare the mechanical strength of the RCa interfaces with both the IST and SST. The five samples included (1, 2, 3, 4 and 6) of comparable dimensions and interfaces were tested. Three hanging points were made in each tendon and in the RCa with 4.0 absorbable braided thread on opposite sides (Fig. 1). The braided thread loops were used to mount the sample in the jaws.

**Fig. 3:**
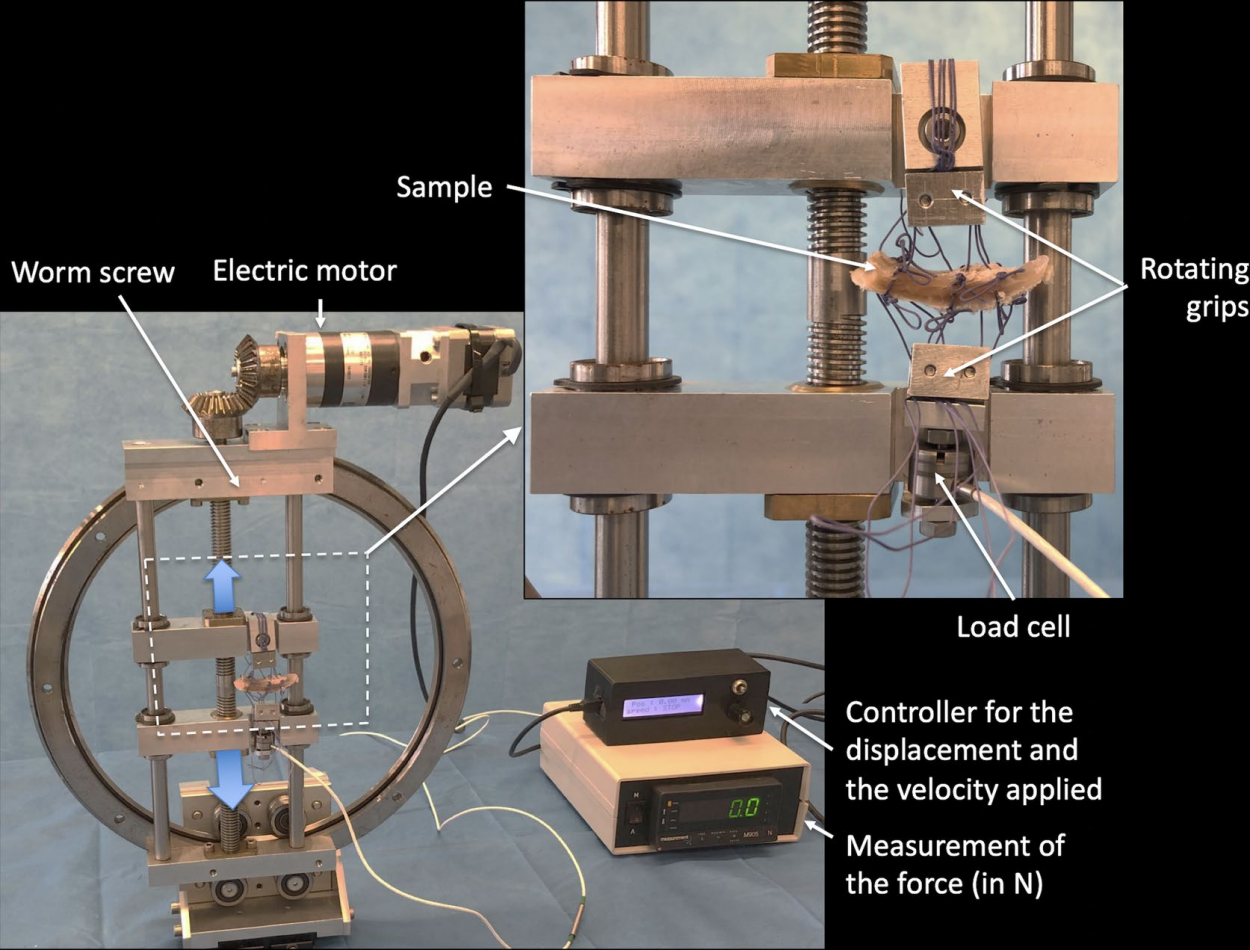
Overview of the experimental setup

An overview of the experimental setup is provided in Fig. 3. It is a home-made device comprising a metal mini-tensile testing machine mounted on a ring to orient the sample in the most suitable direction.

The two jaws were moved in translation by an electric motor and a worm screw, which enabled us to stretch the sample symmetrically. This provided a motionless sample device, which was more suitable for observing the interface area during stretching. The braided threads used for the sutures were fixed in the rotating jaws. Jaw rotation enabled the braided threads to self-balance. The resulting force was measured with a 250 N capacity load cell mounted on the lower jaw. The mechanical load applied corresponded to monotonic uniaxial tensile loading until failure. It was applied with a displacement controller. The load rate was equal to 1.8 mm/min.

## Results

Of the 5 healthy shoulders included (5 right; 1 woman and 4 men; mean age 82.6 ± 4.2 years), all—i.e. the 5 SST-RCa interfaces and the 5 IST-RCa interfaces for samples 1, 2, 3, 4 and 6—were analyzed for the histological study whereas for the biomechanical study only 2 SST-RCa interfaces (for samples 1 and 6) and 3 IST-RCa interfaces (for samples 2, 3 and 4) were analyzed due to suboptimal sample quality and a lack of tissue for adequate suture fixation.

### Histological study

**Fig. 4:**
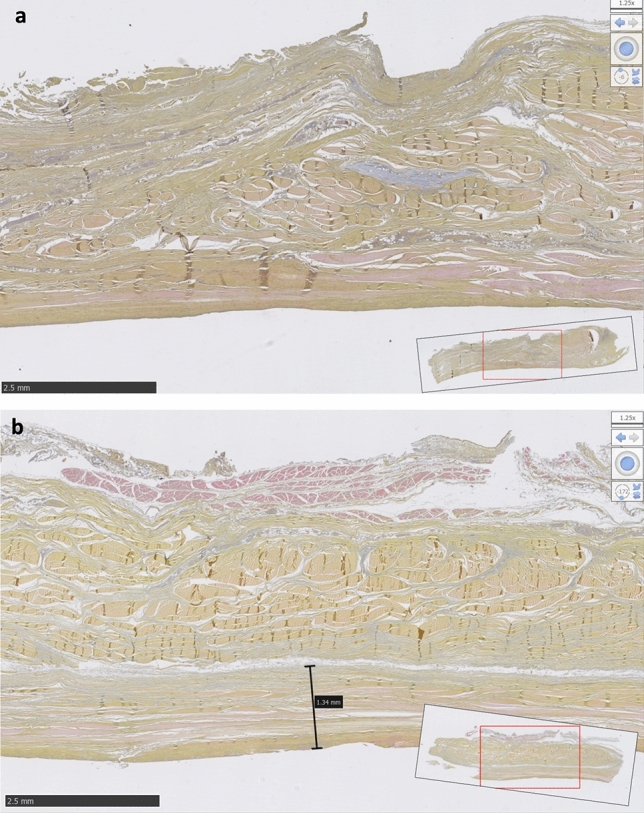
Histological sections made vertically through the rotator cuff and capsule of the shoulder of sample 1 to compare the interface of the SST (**a**) and IST (**b**) tendons with the RCa (semicircular humeral ligament)

The average thickness of the semicircular humeral ligament (RCa) was 1.44 ± 0.20 mm. The histological sections revealed a loose connective tissue layer at the IST-RCa interface, a finding not observed at the SST-RCa interface (Fig. 4).

### Image processing

The detection algorithm implemented allowed the number of areas without horizontal patterns detected and their position to be visualized. Depending on the result, it was possible to produce an image highlighting the borderline position of the semicircular humeral ligament. The final stage of this procedure has yet to be automated.

**Fig. 5:**
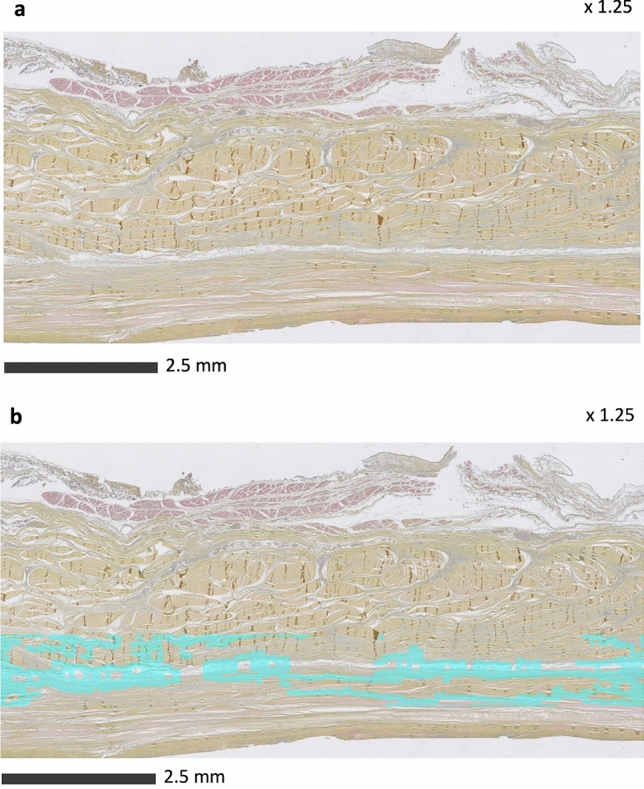
Histological section made vertically through the rotator cuff and capsule of the shoulder of sample 1 to study the interface of the IST with the RCa (semicircular humeral ligament) before (**a**) and after image processing (**b**)

The image in Fig. 5 and Supplemental Materials show that the algorithm can detect the area of loose connective tissue only at the IST-RCa interface in all samples and that the average thickness of the RCa was 0.50 ± 0.20 mm.

### Biomechanical study

**Fig. 6:**
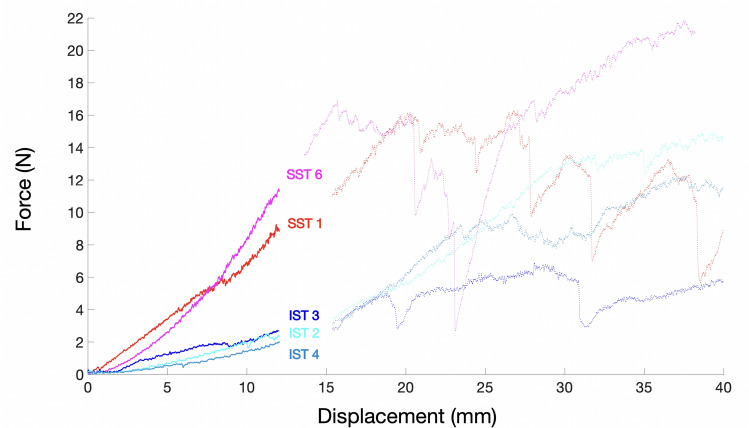
Force–displacement curves for the five prepared samples: 2 SST-RCa interfaces (SST1, SST6) and 3 IST-RCa interfaces (IST2, IST3, IST4). The unblurred area is considered for characterizing the mechanical properties. The blurred area illustrates the chaotic nature of the mechanical response once the damage has started

This section presents the results of the biomechanical analysis in terms of force versus displacement for the five prepared samples: 2 SST-RCa interfaces (samples 1 and 6) and 3 IST-RCa interfaces (samples 2, 3 and 4) (Fig. 6). From a certain level, a decrease in force was observed regardless of the sample considered, which is typically due to damage within the material itself or at the boundaries where the force is applied, in this case the sutures.

As the test is performed at a constant displacement rate, the variation in force may be considered as an indicator of interface rigidity: the higher the force, the higher the interface rigidity. For a sake of simplicity, the force–displacement response has been modeled as a linear relationship, corresponding to the median line between the two areas represented by the dotted lines. The rigidity of the SST-RCa interface was equal to 72.10^–2^ N/mm, which is 4.5 times higher than for the IST-RCa interface (16.10^–2^ N/mm). Moreover, the average maximum forces were 19.0 N and 10.6 N in the SST-RCa and IST-RCa interfaces, respectively. Lastly, the IST-capsule interface ruptured much more quickly and is thus much more vulnerable than the SST-capsule interface.

**Fig. 7:**
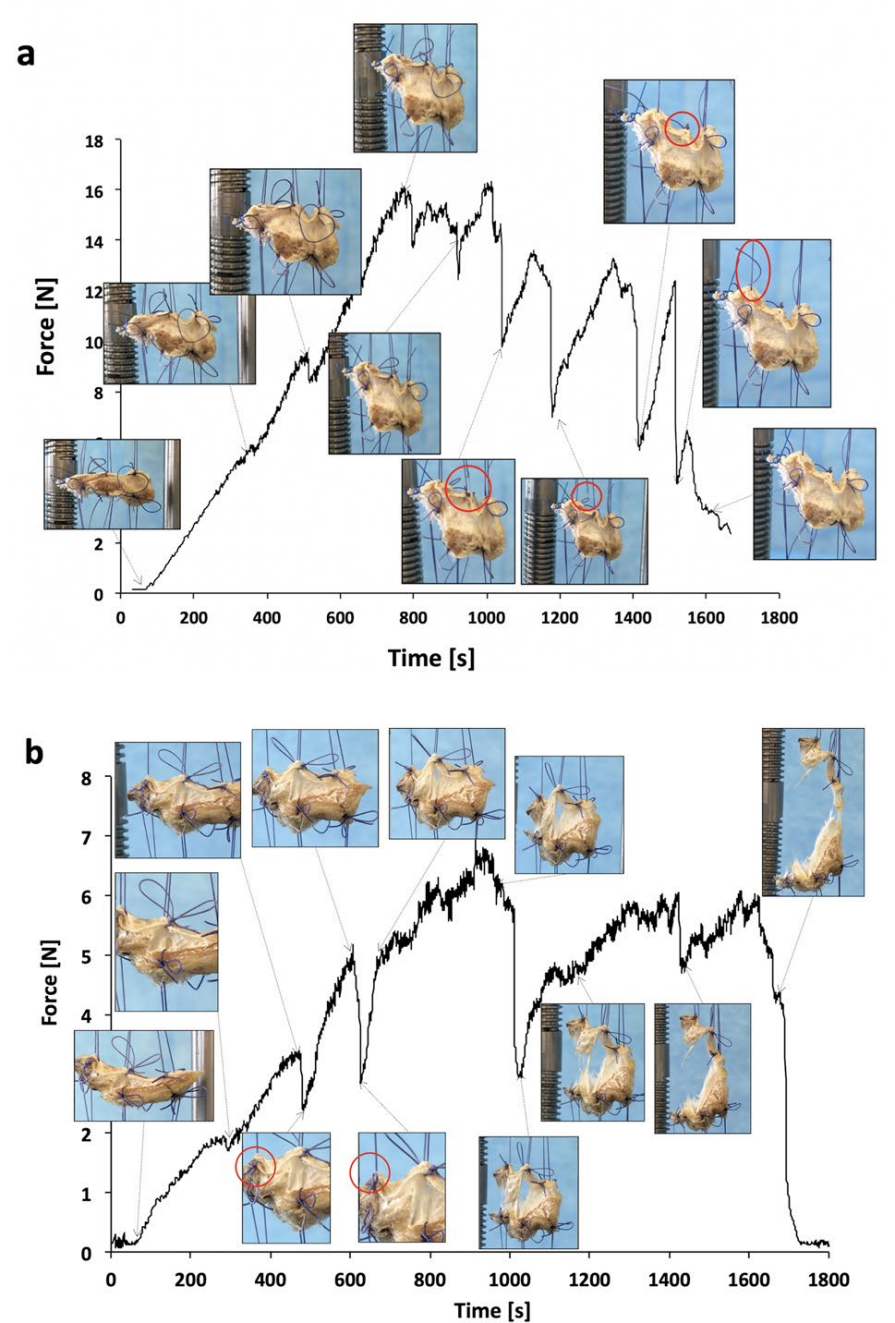
Force versus time in the case of SST-RCa interface of sample 1 (SST1) (**a**) and IST-RCa interfaces of sample 4 (IST4) (**b**). The images illustrate the interface morphology corresponding to each decrease in the force measured during stretching

In addition, Figs. 7a, b provide images of the stretched SST and IST samples at different times, respectively. In the case of the SST (Fig. 7a), the interface resisted the applied force and the sutures failed (see for instance the encircled areas in the images) which correlates well with the results described above.

## Discussion

In the present study, the differences between the interface of the IST and SST with the semicircular humeral ligament or RCa were investigated with anatomical, histological and biomechanical characterization. In the histological study, we showed that there is a loose connective tissue layer located at the IST-RCa interface contrary to the SST-RCa interface. In parallel, two different groups were identified for the mechanical response. The IST-RCa interface group had less rigidity and ruptured much more quickly than the SST-RCa interface, therefore emerging as the most vulnerable interface.

To our knowledge, the authors of a few studies [4, 8, 11, 14, 20] focused on the interface of the rotator cuff tendons with the RCa. This is therefore the first study to report quantitative results on the physics of the IST and SST-RCa interface and provide repeatable results, morphologically—first macroscopically and then on a microscopic scale—and finally biomechanically. In addition, our biomechanical study was conducted with a home-made, dedicated, portable machine, which was lightweight, reliable, and well suited to anatomy laboratory conditions. We also developed a modeling technique for the physical characterization of interfaces to further understanding of the rupture and cracking mechanisms of the SST-RCa and IST-RCa interfaces, which are very different biomechanically, and present in very small volumes.

The mechanical properties of the interface between the rotator cuff tendons and the semicircular humeral ligament were characterized by a dedicated mechanical test. The mechanical response of the interfaces was quantified in terms of force versus displacement and enabled us to determine the maximum forces reached before the onset of interface damage as well as interface rigidity. From a biomechanical perspective, for the same force applied, the interface between the SST and the capsule suffers far less deformation. This result is the first to corroborate the finding by Rouvière [15] and is consistent with the observations of Clark et al*.* [6].

Through our combination of different characterization methods, we showed that the connective tissue layer located at the IST-RCa interface may be considered as loose connective tissue, in line with the observations of Rouvière [15] and Clark [6].

Our experimental observations could explain the clinical or 2D (MR imaging, MR and CT arthrography) radiological extension of SST tears along the IST interface between the tendon and capsule while adherence at this interface appears weak at the IST insertion site [5, 16, 17]. The issue may also be uneven distribution of forces constraining the semicircular ligament and representing the real explanation behind rotator cuff tear genesis.

Furthermore, the semicircular humeral ligament—or RCa—is a crescent of capsular fibers running perpendicular to the axis of the SST and IST extending within the capsule from the intertubercular groove anteriorly to the posterior aspect of the greater tubercle posteriorly [12, 13] and, in our study, the RCa thickness measurements were correlated with those reported in the literature [10, 13]: the average thickness of 1.44 ± 0.20 mm found in our study was similar to the 1.5 ± 0.7 mm demonstrated in a study of 25 normal shoulders by Podgórski et al*.* [13].

Limitations of the study include the low number of subjects as only 5 healthy rotator cuffs were finally exploitable. All samples showed, however, similar findings leading to the same conclusions.

Another limitation of the study lays in the fact that, as formalin fixed tissues may be stiffer and less elastic than living tissue, biomechanical datas obtained in vitro may not be transposable in vivo. Concurrently, histological findings are not altered by such a fixation and presence of loose connective tissue at the IST-RCa interfaces in all subjects, while it is absent at the SST-RCa, supports the reliability of mechanical findings.

Even though the tensile test performed may introduce variability, the mechanical responses were very similar within the same group, which made a quantitative analysis of the results possible.

In the future, precise preliminary measurements with a larger cohort of samples prior to the biomechanical study could improve quantification of the deformed zones after mechanical stretching using the same portable machine. This would be an important step forward in improving understanding of the pathophysiology of basic degenerative lesions and help to provide better treatment. Looking forward, this study raises the question of the behavior of this interface during degenerative rotator cuff tears.

## Conclusion

The IST-RCa interface consists of a loose connective tissue layer contrary to the SST-RCa interface. In parallel, two different groups in terms of the mechanical response were identified: the IST-RCa interface group had less rigidity and ruptured more quickly than the SST-RCa interface, therefore emerging as the most vulnerable interface and explaining a potential extension of rotator cuff tears.

## Supplementary Information

Below is the link to the electronic supplementary material.

Supplementary file1 Fig. E1 Histological section made vertically through the rotator cuff and capsule of the shoulder of the 5 sample 1 to study the interface of the IST with the RCa (semicircular humeral ligament) after image processing with the native image in the upper-right corner. (TIFF 105477 KB)

## Data Availability

All data that support the findings of the current study are accessible upon reasonable request from the corresponding author. No datasets were generated or analysed during the current study.

## Code availability

Available on request.
